# Health Technologies and Impermissible Delays: The Case of Digital Breast Tomosynthesis

**DOI:** 10.1007/s11948-025-00535-2

**Published:** 2025-05-07

**Authors:** Simon Rosenqvist, Magnus Dustler, Johan Brännmark

**Affiliations:** 1 Department of Philosophy, Linguistics and Theory of Science, University of Gothenburg, Box 200, Göteborg, 405 30 Sweden; 2 Diagnostic Radiology, Department of Translational Medicine, Lund University, Malmö, 205 02 Sweden; 3 Medical Radiation Physics, Department of Translational Medicine, Lund University, Malmö, 205 02 Sweden; 4 Department of Philosophy, Stockholm University, Universitetsvägen 10D, Stockholm, 106 91 Sweden

**Keywords:** Health technologies, Health technology assessments, Mammography, Moral principles, Clinical utility, Medical ethics

## Abstract

This paper argues that we have a moral obligation to implement certain health technologies even if we have limited or incomplete evidence of their effectiveness. The focus is on technologies used in non-emergency settings, as opposed to “exceptional cases” such as compassionate use and emergency approvals during public health emergencies. A broadly plausible moral principle – the Ecumenical Principle – is introduced and applied to a test case: the use of Digital Breast Tomosynthesis in mammographic screening. The paper concludes by exploring the implications of the Ecumenical Principle for the adoption of other new health technologies.

## Introduction

When we evaluate a new health technology – such as a surgical procedure or medical device – we normally ask whether its use is morally permissible. In comparison, we rarely ask whether its use is morally required. Such a one-sided focus on moral permissibility may lead decision-makers astray. For example, a decision-maker might correctly determine that a health technology faces no “ethical problems” and that it is thereby morally permissible to implement, but then fail to ask whether its use is *merely* permissible or *also* morally obligatory. In the latter case, even delays in implementing the technology would be impermissible.

Over the following pages, we argue that for certain technologies we have a moral obligation to implement them even if we have only limited or incomplete evidence of their effectiveness. Our argument focuses not on familiar “exceptional” cases for which such obligations can arise, such as compassionate use with respect to medical drugs or emergency approvals in public health emergencies. Instead, we discuss the implementation of less prominent technologies in non-emergency settings, which we illustrate by investigating a novel imaging technology in mammography.

The paper is organized as follows: First, we examine the broader context of our discussion and explain why decision-makers must be watchful of implementing new health technologies too late (Sect. “Moral Obligations and Health Technologies”). Next, we propose a sufficient condition for when we are morally required to implement health technologies, which we call the Ecumenical Principle (Sect. “The Ecumenical Principle”). To demonstrate the principle’s usefulness, we apply it to a concrete test case: the implementation of Digital Breast Tomosynthesis (DBT) in mammographic screening (Sects.“Digital Breast Tomosynthesis”,“Is DBT Clinically Beneficial?”,and“DBT: Justifying Delays”). Our discussion about DBT makes up the core of the paper and draws on cross-disciplinary collaboration between researchers in ethics and diagnostic radiology. In the closing section, we explore how our conclusions regarding DBT can be extended to other health technologies with similar characteristics (Sect. “Beyond DBT”).

## Moral Obligations and Health Technologies

**Fig. 1:**
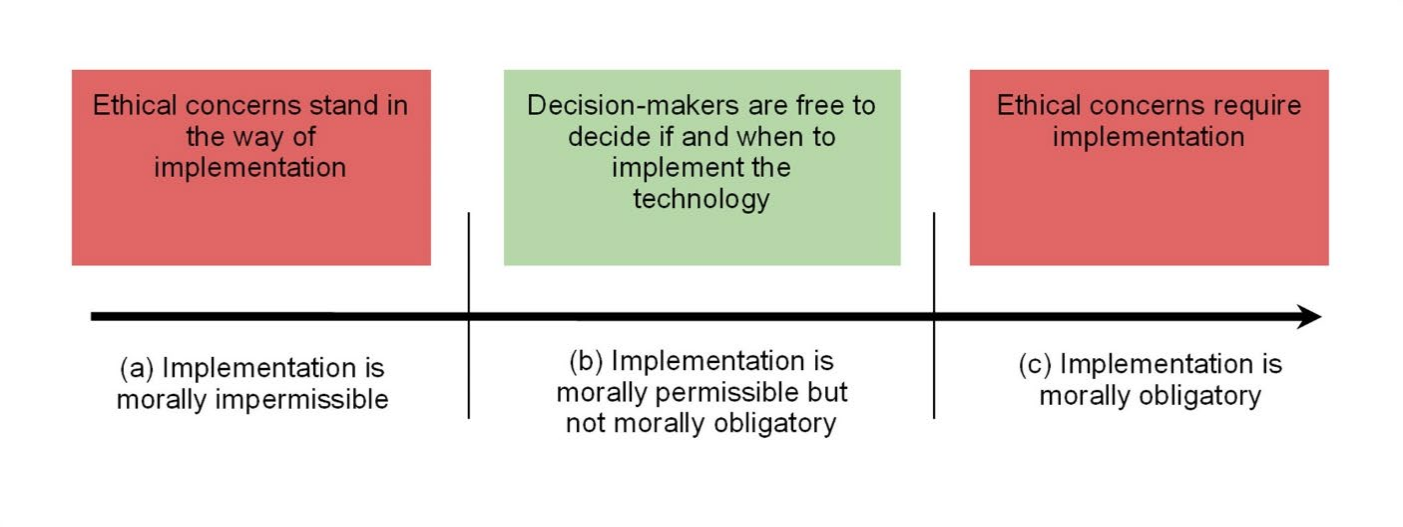
Three categories for the ethical evaluation of health technologies

Health technologies are “the application of organized knowledge and skills in the form of medicines, medical devices, vaccines, procedures and systems” which are “developed to solve a health problem and improve quality of life” (WHO, 2023). On a standard schema for how ethical theories or frameworks sort actions, health technologies fall into three categories: (a) those that are morally impermissible to implement, (b) those that are morally permissible but not morally obligatory to implement, and (c) those that are morally obligatory to implement.^1^ Crucially, it is only for technologies that belong to category (b) that decision-makers are free to decide if and when to implement them, as is illustrated in Fig. 1.

It follows from this categorization that decision-makers can err in two ways when implementing new health technologies. First, they may implement a technology because they believe its use is morally permissible or obligatory, even though it is in fact impermissible. Second, they may fail to implement a technology because they believe its use is morally impermissible or merely permissible, even though it is in fact obligatory. In this paper, we focus on the second type of implementation error, which we believe has received insufficient attention – that of *impermissible delays*. Impermissible delays occur when decision-makers decline to implement a new health technology whose use is presently morally obligatory, perhaps to wait for additional evidence about safety and effectiveness. Importantly, delays are not morally neutral: a decision to not implement a new technology is a decision to keep using existing technologies, and that decision can have serious moral implications.

It is already well-recognized that once we have overwhelming evidence that a health technology is safe and effective, we may be morally required to use it promptly. For example, hospitals are required to maintain an aseptic environment in their operating rooms, as this is widely known to reduce the risk of secondary infection. Moreover, even when the evidence is less compelling, it is sometimes thought that moral obligations can arise in exceptional circumstances such as in public health emergencies (e.g., the deployment of a promising but experimental vaccine in a pandemic) or in individual health emergencies (e.g., providing access to experimental treatments through compassionate use programs). However, there is a blind spot here: cases where our evidence is similarly limited, but which lack the urgency of public or individual emergencies. In such cases, delays can appear innocent and permissible, especially if we are expecting better evidence down the road. At the same time, they can have very serious implications, it is just that their moral importance is not as easily recognized – perhaps because the consequences are hidden downstream in the health-care system, as in the case of preventive healthcare or routine screening.

The concern behind our proposal is that ethical analyses of health technologies end up too strongly focused on looking for reasons *not* to implement or on reasons to delay implementation. Indeed, one recent (and in other respects promising) framework for integrating ethics in health technology assessments illustrates this point, focusing on evaluative tasks such as to “[r]ecognize potentially relevant ethical problems and solutions” and to “list ethical issues around the technology” (Assasi et al., 2016, p. 6). These are important tasks, but not ones that will help us decide between an implementation being merely permissible and being obligatory. If we think of ethical concerns in this way, as mainly providing potential *obstacles* for implementation, we might not take seriously the risk of postponing an implementation for too long. And this is a problem. Our argument therefore has implications not just for decision-makers in the healthcare sector, but also for ongoing research into how to integrate ethics in health technology assessments (e.g., Have, 2004; Hofmann, 2008; Duthie & Bond, 2011; Bellemare et al., 2018; Vandemeulebroucke et al., 2022).

## The Ecumenical Principle

We next propose a framework for structuring ethical analyses, which we call “ecumenical” because it can be accepted by adherents of different normative theories in the domain of health technologies. It identifies concerns that both deontologists and consequentialists tend to emphasize in actual practice, setting aside their theoretical dispute about what ultimately matters.

In moving towards an ecumenical framework for assessing health technologies, we find it reasonable to start with a strongly shared core, and we take that to be *benefit to an individual in the clinical context*. In our proposal, we will refer to benefits in a clinical context using the familiar concept of *clinical utility*, a concept that is “widely used in medicine to describe the relevance and usefulness of an intervention in patient care” and where utility is “the personal benefit that someone has from an intervention, outcome, product, or process” (Lesko et al., 2010, p. 729). For the sake of clarity, here are the key points about our use of this concept in the present context.

First, we consider the clinical utility of a technology as *restricted* to “clinically relevant” effects on beneficiaries. Paradigmatic examples of clinically relevant effects would be increasing the quality of life (or the length of a life worth living) of a patient through medical treatment at a hospital.

Second, the clinical utility of a technology is *comparative*: it is about being better or worse for individuals, rather than being good or bad in an absolute sense. We will assume that the relevant comparison is to currently used health technologies, rather than the absence of any intervention whatsoever.

Third, we assume the most relevant notion when assessing new health-technologies is the technology’s *expected* clinical utility rather than its *actual* clinical utility. The latter involves benefits that individuals really obtain from something, whereas the former is adjusted for subjective uncertainty – i.e., in light of evidence about potential outcomes and the probabilities assigned to them. In one sense, actual utility is what really matters. However, when considering whether to implement a new health technology, it is more reasonable to focus on expected utility, since we cannot know actual utility in advance.

Finally, we will let clinical utility refer to the all-things-considered clinical utility of an implementation. For brevity’s sake, we use the phrase “clinically beneficial” to refer to technologies that have positive clinical utility in this sense.

What role should clinical utility play in assessing moral permissibility and obligations? To begin with, which is important for an ecumenical approach, clinical utility is something that both consequentialists and deontologists should be onboard with as being an undeniable ethical concern.^2^ This is true even if clinical utility is not the *only* thing that matters in an ethical evaluation. For example, deontologists emphasize how there are constraints on our pursuit of utility, such as respect for autonomy, and consequentialists hold that total overall utility is what ultimately matters, which includes clinically irrelevant utility such as the social or political effects of using the technology. Even so, to assess a technology’s clinical utility is a useful starting point – if something lacks even clinical utility, there is normally no need to proceed any further.^3^ Moreover, even an approach that focuses on this limited but shared commitment to clinical utility can generate interesting conclusions, as we hope to show.

The principle that we will propose follows from two plausible claims about clinical utility. According to the first, we always have *some* reason to implement a clinically beneficial technology, because of its expected benefits to patients. In slogan form, we can say that *patient benefits always matter morally*. Or, more precisely:

**C1**: To the extent that a health technology T is clinically beneficial, we have proportionally strong moral reasons to implement T.

Claim C1 is a claim about what moral reasons we have for implementing a health technology. Notably, it is not a claim about whether we ought to implement it, as it says nothing about the deontic status of this act (i.e., whether the act is morally impermissible, permissible, or obligatory). To move from recognizing moral reasons to concluding that we have an obligation, we need an additional claim that “links” the reasons and deontic status. Our suggestion starts from the observation that when we have a moral reason for doing something, and absolutely no reason against doing it, we may not just say “so what?” and refrain from acting. Unlike non-moral reasons, moral reasons may not be *arbitrarily* ignored. This means that for positive clinical utility *not* to settle matters in a particular case, there always needs to be some other consideration pointing in the opposite direction, such as a worry about harm, costs, or the consequences for society more generally. The following claim therefore strikes us as both plausible and sufficiently ecumenical:

**C2**: If we have moral reasons to implement a health technology T, and lack equally strong or stronger reasons to not implement T, we thereby have a moral obligation to implement T.

Jointly, claims C1 and C2 imply:

**The Ecumenical Principle**

We have a moral obligation to implement a health technology T if

- T is clinically beneficial, and
- we have insufficiently strong reasons against implementing T.

This principle is “ecumenical” because it is compatible with a range of moral theories, including consequentialist and deontological ones. It is not meant as a fundamental moral principle, but as a mid-level principle that is useful in decision-making. As such it is similar in function and scope to other mid-level principles, like Beauchamp and Childress’ four principles of biomedical ethics, as well as versions of the “precautionary principle” and the “principle of proportionality” (Beauchamp & Childress, 2019; Rechnitzer, 2024; Hermerén, 2012).

How can the Ecumenical Principle be useful in decision-making? One key thing about fundamental moral principles is that they often do not provide guidance on how to deal with the possibility of delaying. They typically identify right- or wrong-making features, but what ought we to do when we are uncertain, or have inconclusive evidence, about whether some of these obtain or not?^4^ The Ecumenical Principle puts clinical utility at the fore of the ethical analysis, one important implication being that delays cannot always be justified by a lack of “good” or conclusive evidence. Rather, for clinically beneficial technologies, the choice of delaying must be positively argued for. It is not just an ethically safe option. Another way of expressing this point is that the principle implies that, per default, the morally responsible approach to clinically beneficial health technologies is to implement them at once, without delays. In a sense, therefore, as soon as we have established that a technology is clinically beneficial, the burden of proof will shift to the task of coming up with arguments against implementation – even if that burden is sometimes easily met, e.g., by pointing to what are very clearly prohibitive public costs.

One might object that in practice this “default stance” does not make a difference, because there are always reasons that oppose an implementation, such as ones relating to duties to avoid harm or the risk of unintended consequences. For this reason, it all comes back to weighing reasons for and against using a technology, and the Ecumenical Principle is not a principle for weighing reasons. However, this would not be correct: there *are* in fact health technologies for which there are few or no such reasons opposing an implementation – even if the evidence favoring them is weak or incomplete. And in these cases, the default stance makes all the difference. For the other cases where we find ourselves with significant reasons that must be weighed against each other, we must resort to further principles, and the Ecumenical Principles should be compatible with many such principles.

What we will do next is to examine a real-world case that we believe exemplifies this lack of significant opposing reasons, namely the implementation of Digital Breast Tomosynthesis (DBT) in mammographic screening. DBT is a good test case for our discussion, because the technology is similar to ordinary mammography (more about that soon) which makes the comparison of clinical utility to the existing alternative straightforward. Moreover, DBT is a technology whose implementation has been widely debated, for although it has arguably been considered clinically beneficial for a long time, there still exists no conclusive evidence for its superiority over ordinary mammography. For our purposes in this paper, the most challenging issue will be to demonstrate that there are few or insignificant moral reasons to delay the implementation of DBT. We leave this issue for Sect. 6, where we consider three main types of reasons that could count against an implementation.

## Digital Breast Tomosynthesis

In 2020, breast cancer caused 685 000 deaths globally (WHO, 2023, July 29). To save more lives, tumors need to be identified as early as possible, and many countries have implemented population-wide screening programs which invite women without prior symptoms to have their breasts examined. A typical screening program invites all women within a certain age-span (e.g.,, between 45 and 74) either yearly or once every two years.

Screening programs usually employ a group of technologies known collectively as “mammography” to search for cancer, using low-energy x-rays to capture images of breast tissue. A mammography examination is brief and takes only a few minutes. The images are examined by specialist radiologists (“readers”) who, if they find potential signs of cancer, will recall the patient for more detailed tests. Recall tests are more comprehensive and usually include additional technologies apart from mammography, such as ultrasound or biopsies. Most recalled women do not have cancer.

Mammography is not a single technology, but several. The most widely used form of mammography today is Digital Mammography (DM), which captures grayscale 2D-images of breast tissue. The use of DM and its predecessor, film-based mammography, have been largely successful at both finding dangerous tumors and ruling out the existence of cancer. In medical terminology, current mammography technology has both a reasonably high sensitivity (i.e., it is good at finding cancer) and specificity (i.e., it is good at determining that no cancer is present). Even so, tumors hidden behind dense obscuring breast tissue sometimes fail to show up on these flat 2D-images. Researchers have therefore developed an alternative form of mammography which makes it possible to examine the breast “in depth.” This technology is known as Digital Breast Tomosynthesis (DBT) (Niklason et al., 1997).

DBT works similarly to DM, but instead of just capturing 2D-images, DBT captures a series of images from different angles and combines them into a pseudo 3D-image. This image shows multiple layers of the breast that readers can “scroll” through, letting them search for tumors that would otherwise be hidden behind overlaying tissue. Large-scale studies have demonstrated that DBT finds a greater number of tumors than DM, while it is comparable when it comes to ruling out the existence of cancer (Ciatto et al., 2013; Bernardi et al., 2016; Zackrisson et al., 2018; Skaane et al., 2019; Heindel et al., 2022). In other words, DBT has higher sensitivity, and its specificity is not worse, compared with DM. The difference in sensitivity is by no means trivial: for example, a recently published randomized study detected 48% more cancers with DBT when compared to DM (Heindel et al., 2022).

Despite the promise of DBT, DM remains the standard technology in screening, and regulatory authorities are divided on how to approach the technology. Since 2011, US health-care providers are permitted to use DBT in screening after a decision by the Food and Drug Administration (FDA). In contrast, European authorities remain cautious. The latest recommendation from the European Commission Initiative on Breast Cancer is “conditional”, which means that countries are recommended to employ one (and only one) of the two technologies, either DBT or DM (ECIBC, 2020).

We will next examine potential motivations behind the more cautious approach to DBT. But first, we will note an interesting fact about the two technologies: DM and DBT can often be implemented using the same imaging equipment. In fact, modern mammography systems are now frequently delivered “DBT-ready”, which means that they can be used for both DM and DBT – even in the same screening session – with similar procedure and minimal delays. This means that some clinics have had the equipment to screen with DBT for years, since they had to buy it to screen with DM, yet do not use this equipment for DBT. These clinics are essentially opting out of discovering potentially dangerous tumors with equipment they have already installed and paid for. This circumstance will be important for our discussion, as in these cases a major reason not to implement DBT (i.e., equipment costs) is absent.

## Is DBT Clinically Beneficial?

We will now argue that it is morally obligatory to use existing DBT-ready equipment for DBT in screening. This argument illustrates how the Ecumenical Principle can be useful for identifying impermissible delays, although if it is successful, it also points to a problematic flaw in the European approach to implementing DBT.^5^ The argument proceeds in two steps. This section argues that DBT is clinically beneficial and therefore satisfies condition (a) of the Ecumenical Principle. The next section argues that the use of DBT with DBT-ready equipment also satisfies condition (b).

It is tempting to think that since DBT has higher sensitivity and no worse specificity compared to the presently used technology (i.e., DM), then it *must* be clinically beneficial – no argument needed. But matters are more complicated. For while it is well established that DBT detects more cancers than DM, it is not known for certain that *treating* these additional cancers will benefit patients. The problem is that we cannot tell harmless and harmful tumors apart, and therefore treat all tumors, even ones that would not have any negative health effects. This is known as overdiagnosis, and it is a problem because treatments for breast cancer – whether necessary or not – often result in significant suffering.

The possibility of overdiagnosis has been cited as a reason to keep using DM in screening, instead of implementing DBT.^6^ Yet it is important to distinguish between the mere possibility of overdiagnosis and actual evidence for it – only the latter is relevant for questions of clinical utility. It is true that a recent meta-study did not find a difference in the effect of DBT on “interval cancer”, which is cancer discovered because of symptoms between screening rounds (Houssami et al., 2021). This is an indication of overdiagnosis, but a weak one, as the included studies were not statistically powered to examine interval cancer, and no studies have been conducted on repeated screening rounds. For all that we know, the effect from DBT could therefore be delayed. More importantly, not only does DBT discover more tumors than DM, but the additionally discovered tumors appear to have similar characteristics to the ones that are discovered with DM, making it very unlikely that they represent a specific subpopulation of harmless cancers (cf. Johnson et al., 2021). In fact, DBT has even been reported to discover *more* aggressive tumors than DM (Conant et al., 2020). On balance, therefore, we believe that DBT should at the present stage be considered clinically beneficial. What the debate about overdiagnosis shows is that our evidence about DBT degree remains incomplete and might change in the future.

## DBT: Justifying Delays

Even if technologies are clinically beneficial, there can be reasons to delay implementation. We suggest that there are three main groups of such potential ethical concerns. First, we have other relevant utilities, apart from clinical utility. These are concerns that a consequentialist will emphasize, but which also matter to deontologists who recognize a duty of beneficence. Second, we have moral concerns apart from utility. These will be highlighted by deontologists, but consequentialists may also find them important because attending to them will lead to better long-term consequences. Finally, there are issues more directly concerned with handling uncertainty and unforeseen consequences, what we may call “precautionary concerns”.

### Wider Utility Concerns

On the ecumenical principle proposed here, clinical utility is the starting-point in an ethical analysis of whether a new health technology should be implemented. Other utilities are also important, but they primarily enter the analysis as possible defeaters of the basic clinical-utility-based reason for implementation. Two such types of consequences that can justify delays to clinically beneficial technologies are *cost effectiveness* and *impact on future scientific progress*.

Cost-effectiveness matters because available resources should be deployed in the best way possible. A technology will usually be considered cost effective if and only if the cost per quality adjusted life year (QALY) does not exceed a predetermined threshold – the “willingness-to-pay threshold”. So far, economic analyses on DBT have been on the fence about its cost-effectiveness – it depends on assumptions about increased costs due to longer reading times, cancer detection, recall rates, as well as the willingness-to-pay threshold (see, for example, Lowry et al., 2020; Sankatsing et al., 2020; Cressman et al., 2021; Wang et al., 2020).

A lack of evidence that a technology is cost-effective is not a reason to delay its implementation. That said, the *risk* that it is not cost-effective might be. From a wider-utility perspective, such a risk might be relevant to consider if an implementation incurs large non-recoverable costs from, for example, purchasing expensive equipment. Delays might also be justified if a technology is hard to de-implement once it is implemented, such as due to psychological biases like the sunk cost fallacy, which might leave us stuck with a less cost-effective technology for the near future. However, neither problem is applicable to the use of DBT with DBT-ready equipment. That is because in this case, the expensive equipment is already paid for and present at the clinics. Thus, if DBT turns out to not be cost-effective in the future, the same equipment could be used for DM instead.

Consider next the impact on scientific progress. Once a technology is introduced to the clinic it may become difficult to conduct some studies. Patients may be reluctant to join control groups, worried about giving up their chance to benefit from the new technology. With less and lower quality evidence that a technology works, future research in the field becomes more complicated. That is because we want to compare newly developed technologies to those currently used, but these comparisons suffer if we have limited evidence for the merits of presently used technologies. Accepting delays in exchange for better evidence, and potentially better long-term consequences, can therefore be permissible.

The use of DBT with DBT-ready equipment might limit research to some extent. Even so, we can still evaluate the sensitivity and specificity of DBT, since the evaluation by readers is distinct from the process of capturing images. For instance, it remains possible to screen with both DM and DBT in screening, and then have one group of readers evaluate the 2D images and another group evaluate the pseudo-3D images. It will be harder to assess the effects on mortality, which require patient groups that use only DM and forego DBT, but we are not aware of any current or planned studies to assess the effects on mortality from the use of DBT, and such studies would require many years or even decades to complete. For this reason, these limitations appear more theoretical than practical.

### Other Moral Concerns

An important ethical concern, distinct from overall utility, has to do with respecting the separateness of persons, a demand that individuals should not summarily be sacrificed for the greater benefit of *other* individuals. This concern is typically voiced by deontologists, although consequentialists often also take it seriously, because of indirect positive consequences, e.g., in designing public institutions that people can trust. Using terminology from Scanlonian Contractualism one might articulate this in terms of how, even when the implementation of a new technology would have positive consequences *on the whole*, those few people who are made worse off by it can still have *reasonable complaints* against its adoption, because *their* interests would not be sufficiently respected in implementing it (Scanlon, 2000). Other relevant concerns here are about equal treatment and non-discrimination, as well as respecting the autonomy of individuals, enabling them to make their own decisions and live their lives according to their own life-plans. Such issues can provide grounds for not implementing a health technology – even when it is both clinically beneficial and cost-effective.

The implementation of DBT would, however, not raise these problems. The concern about individuals being sacrificed for the greater good is only relevant when patients are made worse off by an implementation, which is not the case for DBT. As for concerns of equality and non-discrimination, the use of DBT is expected to benefit women with dense breasts more than other women. But those benefits can be said to increase rather than decrease overall equality, since they occur because of how the current screening practice (i.e., DM) is less adequate for women with denser breasts than for other women.^7^ An implementation of DBT might therefore be seen as addressing an existing inequality, making the screening of women with dense breasts more on a par in quality with the screening of other women. Finally, as for autonomy, DBT can be implemented without changing anything significant to existing processes used for DM, such as how to formulate screening invitation letters to women.

### Precautionary Concerns

Implementing new technologies can have severe and adverse consequences that are difficult to foresee, either because we lack information about which more specific outcomes may follow a decision, or because we lack information about which probabilities should be assigned to outcomes. Such consequences will be difficult to analyze, whether from a consequentialist or deontological perspective. In some such circumstances, one may therefore hold that a version of the precautionary principle – or precautionary concerns more generally – gives us reason to delay the implementation of a clinically beneficial technology.

We cannot here go into the large literature on precaution and how to best understand the precautionary principle (see, e.g., Hansson, 1997; Gardiner, 2006; John, 2007 for some attempts, and Rechnitzer, 2024 for an overview). However, we believe that any plausible precautionary approach is unlikely to apply to the use of DBT with DBT-ready equipment. That is because DBT and DM carry the same type of risks, and since we have no reason to believe that the use of DBT would result in an especially severe outcome when compared to DM. The implementation of DBT is in this sense different from, for example, the introduction of novel therapeutic drugs, where decade-long delays can sometimes be justified on precautionary grounds. Moreover, even if a version of the precautionary principle *does* apply to this case it is not clear whether it supports DM or DBT. For the precautionary principle is not married to the status quo, and one could also use it to argue that we should implement DBT swiftly, so that we do not leave large numbers of potentially dangerous cancers undetected.

## Beyond DBT

In light of the above discussion, we suggest the use of DBT in screening with existing DBT-ready equipment satisfies both conditions of the Ecumenical Principle and is therefore morally required. It follows that delaying the implementation of DBT in these circumstances is impermissible. We also submit that this result is not obvious or trivial – as we have pointed out, there is still no consensus on the use of DBT even with preexisting equipment.

Even if cases like that of DBT turn out to be rare, our discussion might have implications for decision-makers, given that they desire to avoid even rare instances of moral wrongdoing. But we also believe our argument may be more broadly applicable. To see this, note that the use of DBT with DBT-ready equipment has features that ensure many “normal” reasons to delay an implementation do not apply, including:

- that DBT is easy and inexpensive to implement and de-implement,
- that it does not require major changes to existing processes and infrastructure,
- that it has well-distributed benefits and risks of harm among screening subjects,
- that its implementation would not significantly impede scientific research on the technology, and,
- that its implementation raises no precautionary concerns.

Where can we find other health technologies that are clinically beneficial and share some or all of these characteristics? Even within the narrow field of breast imaging, there are other examples of such technologies. For instance, when DM replaced film-based mammography the situation was in many respects like the shift from DM to DBT: digital mammography was a promising technology, but it was not known for sure whether it would prove superior to film-based mammography. Today, mammography faces similar questions about the use of AI-assisted reading, where specialized software complements the role of human readers in examining mammographic images (Lång et al., 2023). Although it is important to beware of more general issues with AI, such as bias due to limited training data, some forms of software assistance are likely to become both modestly supported and have few drawbacks to their use, while having the same “easy to implement, easy to de-implement” character as DBT. It is easy to see that using such a technology could be permissible, especially if it arrives pre-installed on new systems. But it is easy to miss that its use might also be morally obligatory, because of the prospective benefits to patients.

Other examples may be found in testing and screening for diseases more generally. Since testing and screening concerns obtaining and processing information about patients, this can allow for the parallel use of more than one technology. One could imagine a promising blood test that can easily and cheaply be added to an existing batch of tests. Such tests might only need modest support to be morally required, given that there are few adverse consequences that could result from their use.

Consider also the implementation of new medical methods and procedures. New procedures may be added on top of existing practices, without introducing new risks to patients. While the costs of disseminating information and retraining should not be discounted, some new procedures might be both easy to implement and easy to get rid of. One example of such a health technology could be the use of checklists in surgery, which has shown great promise to reduce errors and improve outcomes for patients (Gawande, 2010). Introducing checklists to operating rooms appears to share many features with technologies like DBT, being cheap and easy to implement and de-implement while raising few or no precautionary concerns.

To be clear, although we have argued that DBT and technologies that fit the same profile may be morally obligatory to implement, we should not ignore the practical challenges, logistical complexities, and systemic implications of such implementations. For each relevant case, a detailed analysis must be made. But to recognize a potential moral mistake – that delays of this type can be impermissible, even in the face of inconclusive evidence – is an important first step along that path. Moreover, since the details of individual cases will often be hard to assess for a casual observer, such analyses should be performed as part of a systematic ethical evaluation. We therefore suggest that frameworks for integrating ethics in health technology assessments are adapted to better help decision-makers identify impermissible delays.

## Footnotes

1 Many ethicists also recognize a fourth category: supererogatory acts, ones that go morally beyond just meeting our obligations. In the three-category schema we use, these would simply count as permissible but not obligatory, which does not fully capture their character. We will however set this complication aside. Paradigmatic cases of supererogation tend to be about individuals sacrificing their own interest for the sake of moral ends, while in a policy context the choice is rather about the best use of all available resources. See Weinberg (2011) for an argument against supererogation as a category for government action.

2 Consequentialists embrace maximizing the good as *the* ethical principle, of course, but most deontologists also see beneficence as an important duty. Kant clearly recognized it, claiming that “the ends of a subject who is an end in itself must as far as possible be also my ends” (Kant, 1785 [1996], 4:430), and for W. D. Ross (2002 [2003]), there is a duty of beneficence, one that “rests on the mere fact that there are other beings in the world whose condition we can make better in respect of virtue, or of intelligence, or of pleasure” (ibid., p. 21). Libertarian rights theorists, such as Nozick (1974), who only recognize negative rights and duties would be an exception, but such a position would require a very different approach to policy-making in a health-care context anyway.

3 There are exceptions – for example, it could sometimes be reasonable to implement a technology which reduces individual patient benefits but that also, perhaps for reasons of cost-effectiveness, can be deployed on a larger scale and reach more beneficiaries.

4 Consequentialism can straightforwardly handle *risk* (in terms of expected utility), but *uncertainty* is a more complicated matter, especially since there might be *unknown unknowns*. Its difficulties in handling such uncertainties could even be an objection to consequentialism as a theory, see Lenman (2000) for a discussion. That said, while precautionary concerns are important, we do not believe these unknown unknowns provide a more principled obstacle for our ethical analysis here.

5 For another discussion of this approach, see Rosenqvist et al. (2024).

6 For example, this was one of the motivations behind the Swedish decision to postpone implementing DBT in screening (Socialstyrelsen, 2023a).

7 This was pointed out in the ethical analysis that accompanied a proposal for new guidelines for the Swedish screening program for breast cancer (Socialstyrelsen, 2023b).

## References

[CR1] Assasi, N., Tarride, J. E., O’Reilly, D., & Schwartz, L. (2016). Steps toward improving ethical evaluation in health technology assessment: A proposed framework. *BMC Medical Ethics*, *17*(1), 1–16. 10.1186/s12910-016-0118-0 27267369 10.1186/s12910-016-0118-0 PMC4895959

[CR2] Beauchamp, T. L., & Childress, J. F. (2019). *Principles of biomedical ethics* (8th edn.). Oxford University Press.

[CR3] Bellemare, C. A., Dagenais, P., -Bédard, K., Béland, S., Bernier, J. P., Daniel, L., Gagnon, C. É., Legault, H., Parent, G. A., M., & Patenaude, J. (2018). Ethics in health technology assessment: A systematic review. *International Journal of Technology Assessment in Health Care*, *34*(5), 447–457. 10.1017/S0266462318000508 30296950 10.1017/S0266462318000508

[CR4] Bernardi, D., Macaskill, P., Pellegrini, M., Valentini, M., Fantò, C., Ostillio, L., Tuttobene, P., Luparia, A., & Houssami, N. (2016). Breast cancer screening with tomosynthesis (3D mammography) with acquired or synthetic 2D mammography compared with 2D mammography alone (STORM-2): A population-based prospective study. *The Lancet Oncology*, *17*(8), 1105–1113. 10.1016/S1470-2045(16)30101-2 27345635 10.1016/S1470-2045(16)30101-2

[CR5] Ciatto, S., Houssami, N., Bernardi, D., Caumo, F., Pellegrini, M., Brunelli, S., Tuttobene, P., Bricolo, P., Fantò, C., Valentini, M., Montemezzi, S., & Macaskill, P. (2013). Integration of 3D digital mammography with tomosynthesis for population breast-cancer screening (STORM): A prospective comparison study. *The Lancet Oncology*, *14*(7), 583–589. 10.1016/S1470-2045(13)70134-7 23623721 10.1016/S1470-2045(13)70134-7

[CR6] Conant, E. F., Samantha, P., Zuckerman, E. S., McDonald, Susan, P., Weinstein, K. E., Korhonen, J. A., Birnbaum, J. D., Tobey, M. D., Schnall, Rebecca, A., & Hubbard (2020). Five consecutive years of screening with digital breast tomosynthesis: Outcomes by screening year and round. *Radiology*, *295*(2), 285–93. 10.1148/radiol.2020191751 10.1148/radiol.2020191751 PMC7193918 32154771

[CR7] Cressman, S., Mar, C., Sam, J., Kan, L., Lohrisch, C., & Spinelli, J. J. (2021). The cost-effectiveness of adding tomosynthesis to mammography-based breast cancer screening: An economic analysis. *CMAJ Open*, *9*(2), E443–E450. 10.9778/cmajo.20200154 10.9778/cmajo.20200154 PMC8101637 33888549

[CR8] Duthie, K., & Bond, K. (2011). Improving ethics analysis in health technology assessment. *International Journal of Technology Assessment in Health Care*, *27*(1), 64–70. 10.1017/S0266462310001303 21262075 10.1017/S0266462310001303

[CR9] ECIBC. (2020). European guidelines on breast cancer screening and diagnosis. Question: Should screening using digital breast tomosynthesis vs. digital mammography be used in organised screening programmes for early detection of breast cancer in asymptomatic women? European Commission Initiative on Breast Cancer (ECIBC). https://healthcare-quality.jrc.ec.europa.eu/sites/default/files/Guidelines/Old%20documents/ECIBC_GLs_DBT_vs_DM_2020.pdf

[CR10] Gardiner, S. M. (2006). A core precautionary principle. *Journal of Political Philosophy*, *14*(1), 33–60. 10.1111/j.1467-9760.2006.00237.x

[CR11] Gawande, A. (2010). *The checklist manifesto*. Profile Books Ltd.

[CR12] Hansson, S. O. (1997). The limits of precaution. *Foundations of Science*, *2*, 293–306.

[CR14] ten Have, H. (2004). Ethical perspectives on health technology assessment. *International Journal of Technology Assessment in Health Care*, *20*(1), 71–76. 10.1017/S0266462304000819 10.1017/s0266462304000819 15176180

[CR15] Heindel, W., Weigel, S., Gerß, J., Hense, H. W., Sommer, A., Krischke, M., & Kerschke, L. (2022). Digital breast tomosynthesis plus synthesised mammography versus digital screening mammography for the detection of invasive breast cancer (TOSYMA): A multicentre, open-label, randomised, controlled, superiority trial. *The Lancet Oncology*, *23*(5), 601–611. 10.1016/S1470-2045(22)00194-2 35427470 10.1016/S1470-2045(22)00194-2

[CR16] Hermerén, G. (2012). The principle of proportionality revisited: Interpretations and applications. *Medicine Health Care and Philosophy*, *15*(4), 373–382. 10.1007/s11019-011-9360-x 22042598 10.1007/s11019-011-9360-x

[CR17] Hofmann, B. M. (2008). Why ethics should be part of health technology assessment. *International Journal of Technology Assessment in Health Care*, *24*(4), 423–429. 10.1017/S0266462308080550 18828936 10.1017/S0266462308080550

[CR18] Houssami, N., Zackrisson, S., Blazek, K., Hunter, K., Bernardi, D., Lång, K., & Solveig Hofvind. (2021). Meta-Analysis of prospective studies evaluating breast cancer detection and interval cancer rates for digital breast tomosynthesis versus mammography population screening. *European Journal of Cancer*, *148*, 14–23. 10.1016/j.ejca.2021.01.035 33706172 10.1016/j.ejca.2021.01.035

[CR19] John, S. D. (2007). How to take deontological concerns seriously in risk-cost-benefit analysis: A re-interpretation of the precautionary principle. *Journal of Medical Ethics*, *33*(4), 221–224. 10.1136/jme.2005.015677 17400621 10.1136/jme.2005.015677 PMC2652780

[CR20] Johnson, K., Lång, K., Ikeda, D. M., Åkesson, A., Andersson, I., & Zackrisson, S. (2021). Interval breast cancer rates and tumor characteristics in the prospective population-based Malmö breast tomosynthesis screening trial. *Radiology*, *299*(3), 559–567. 10.1148/radiol.2021204106 33825509 10.1148/radiol.2021204106

[CR21] Kant, I. (1785 [1996]). *Groundwork of the metaphysics of morals, trans. Mary Gregor, in the Cambridge edition of the works of Immanuel Kant: Practical philosophy*. Cambridge University Press.

[CR25] Lång, K., Josefsson, V., Larsson, A. M., Larsson, S., Högberg, C., Sartor, H., Hofvind, S., Andersson, I., & Rosso, A. (2023). Artificial intelligence-supported screen reading versus standard double reading in the mammography screening with artificial intelligence trial (MASAI): A clinical safety analysis of a randomised, controlled, non-inferiority, single-blinded, screening accuracy study. *The Lancet Oncology*, *24*(8), 936–944. 10.1016/S1470-2045(23)00298-X 37541274 10.1016/S1470-2045(23)00298-X

[CR22] Lenman, J. (2000). Consequentialism and cluelessness. *Philosophy & Public Affairs*, *29*(4), 342–370. 10.1111/j.1088-4963.2000.00342.x

[CR23] Lesko, L. J., Zineh, I., & Huang, S. M. (2010). What is clinical utility and why should we care? *Clinical Pharmacology & Therapeutics*, *88*(6), 729–733. 10.1038/clpt.2010.229 21081937 10.1038/clpt.2010.229

[CR24] Lowry, K. P., Trentham-Dietz, A., Schechter, C. B., Alagoz, O., Barlow, W. E., Burnside, E. S., Conant, E. F., Hampton, J. M., Huang, H., Kerlikowske, K., Lee, S. J., Miglioretti, D. L., Sprague, B. L., Tosteson, A. N. A., Yaffe, M. J., & Stout, N. K. (2020). Long-term outcomes and cost-effectiveness of breast cancer screening with digital breast tomosynthesis in the United States. *Journal of the National Cancer Institute*, *112*(6), 582–589. 10.1093/jnci/djz184 31503283 10.1093/jnci/djz184 PMC7301096

[CR26] Niklason, L. T., Christian, B. T., Niklason, L. E., Kopans, D. B., Castleberry, D. E., Opsahl-Ong, B. H., Landberg, C. E., Slanetz, P. J., Giardino, A. A., Moore, R., Albagli, D., DeJule, M. C., Fitzgerald, P. F., Fobare, D. F., Giambattista, B. W., Kwasnick, R. F., Liu, J., Lubowski, S. J., Possin, G. E., & Wirth, R. F. (1997). Digital tomosynthesis in breast imaging. *Radiology*, *205*(2), 399–406. 10.1148/radiology.205.2.9356620 9356620 10.1148/radiology.205.2.9356620

[CR27] Nozick, R. (1974). *Anarchy, state, and utopia*. Basic Books.

[CR28] Rechnitzer, T. (2024). Precautionary principles. In *Internet encyclopedia of philosophy*. https://iep.utm.edu/pre-caut/

[CR41] Rosenqvist, S., Brännmark, J., & Dustler, M. (2024). Digital breast tomosynthesis in breast cancer screening: an ethical perspective. *Insights into Imaging*, *15*(1), 1–5. 10.1186/s13244-024-01790-w 10.1186/s13244-024-01790-w PMC11347518 39186168

[CR29] Ross, W. D. (2002). *The right and the good*. Philip Stratton-Lake (Ed., new edn.). Clarendon Press.

[CR30] Sankatsing, V. D. V., Juraniec, K., Grimm, S. E., Joore, M. A., Pijnappel, R. M., de Koning, H. J., & van Ravesteyn, N. T. (2020). Cost-effectiveness of digital breast tomosynthesis in Population-based breast cancer screening: A probabilistic sensitivity analysis. *Radiology*, *297*(1), 40–48. 10.1148/radiol.2020192505 32749212 10.1148/radiol.2020192505 PMC7526946

[CR31] Scanlon, T. M. (2000). *What we owe to each other*. Belknap Press of Harvard University.

[CR34] Skaane, P., Bandos, A. I., Niklason, L. T., Sebuødegård, S., Østerås, B. H., Gullien, R., Gur, D., & Hofvind, S. (2019). Digital mammography versus digital mammography plus tomosynthesis in breast cancer screening: The Oslo tomosynthesis screening trial. *Radiology*, *291*(1), 23–30. 10.1148/radiol.2019182394 30777808 10.1148/radiol.2019182394

[CR32] Socialstyrelsen (2023a). Screening för bröstcancer—Socialstyrelsens rekommendation—Slutversion (2023-5-8564). Socialstyrelsen.

[CR33] Socialstyrelsen (2023b). Översyn av rekommendation om screening för bröstcancer—Etisk bilaga (2023-2-8360 - bilaga 2). Socialstyrelsen.

[CR35] Vandemeulebroucke, T., Denier, Y., Mertens, E., & Gastmans, C. (2022). Which framework to use? A systematic review of ethical frameworks for the screening or evaluation of health technology innovations. *Science and Engineering Ethics*, *28*(3), 26. 10.1007/s11948-022-00377-2 35639210 10.1007/s11948-022-00377-2

[CR36] Wang, J., Phi, X. A., Greuter, M. J. W., Daszczuk, A. M., Feenstra, T. L., Pijnappel, R. M., Vermeulen, K. M., Buls, N., Houssami, N., Lu, W., & de Bock, G. H. (2020). The cost-effectiveness of digital breast tomosynthesis in a population breast cancer screening program. *European Radiology*, *30*(10), 5437–5445. 10.1007/s00330-020-06812-x 32382844 10.1007/s00330-020-06812-x PMC7476964

[CR37] Weinberg, J. (2011). Is government supererogation possible? *Pacific Philosophical Quarterly*, *92*(2), 263–281. 10.1111/j.1468-0114.2011.01392.x

[CR38] World Health Organisation (2023, July 12). Breast cancer. Retrieved July 12, 2023, from https://www.who.int/news-room/fact-sheets/detail/breast-cancer

[CR39] World Health Organisation (2023, July 29). Health Technology Assessment. Retrieved July 29, 2023, from https://www.who.int/teams/health-product-policy-and-standards/assistive-and-medical-technology/medical-devices/assessment

[CR40] Zackrisson, S., Lång, K., Rosso, A., Johnson, K., Dustler, M., Förnvik, D., Förnvik, H., Sartor, H., Timberg, P., Tingberg, A., & Andersson, I. (2018). One-view breast tomosynthesis versus two-view mammography in the Malmö breast tomosynthesis screening trial (MBTST): A prospective, population-based, diagnostic accuracy study. *The Lancet Oncology*, *19*(11), 1493–1503. 10.1016/S1470-2045(18)30521-7 30322817 10.1016/S1470-2045(18)30521-7

